# Chemical chaperone rescue of mutant human cystathionine β-synthase

**DOI:** 10.1016/j.ymgme.2007.04.011

**Published:** 2007-08

**Authors:** Laishram R. Singh, Xulin Chen, Viktor Kožich, Warren D. Kruger

**Affiliations:** a Division of Population Science, Fox Chase Cancer Center, 333 Cottman Avenue, Philadelphia, PA 19111, USA; b Institute of Inherited Metabolic Disease, 1st Faculty of Medicine, Charles University, Prague, Czech Republic

**Keywords:** Homocystinuria, Methionine metabolism, Osmolytes, Chaperone, Mutation

## Abstract

Missense mutations in the cystathionine beta-synthase (*CBS*) gene, such as I278T, are responsible for CBS deficiency, the most common inherited disorder in sulfur metabolism. Expression of human mutant CBS proteins in *Saccharomyces cerevisiae* reveals that most disease causing mutations severely inhibit enzyme activity and cannot support growth of yeast on cysteine-free media. Here, we show that the osmolyte chemical chaperones glycerol, trimethylamine-*N*-oxide, dimethylsulfoxide, proline or sorbitol, when added to yeast media, allows growth on cysteine-free media and causes increased enzyme activity from I278T and three other mutant CBS proteins. Rescuable mutants are ones that are predicted to cause a decrease in solvent accessible surface area. The increase in enzyme activity is associated with stabilization of the tetramer form of the enzyme. This effect is not specific to yeast, as addition of the chaperone glycerol resulted in increased I278T activity when the enzyme is produced either in *Escherichia coli* or in a coupled *in vitro* transcription/translation reaction. However, no stimulation of specific activity was observed when chaperones were added directly to purified I278T indicating that the presence of chemical chaperones is required during translation. We also found that by mixing different chaperones we could achieve rescue at significantly lower chaperone concentrations. Taken together, our data show that chemical chaperones present during the initial folding process can facilitate proper folding of several mutant CBS proteins and suggest it may be possible to treat some inborn errors of metabolism with agents that enhance proper protein folding.

## Introduction

Missense mutations, genetic alterations that result in the production of proteins with single amino acid changes, are the most common type of mutation found in inborn errors of metabolism. For example, they make up 62.4% of the mutant alleles described in phenylketonuria patients, 65% of the alleles seen in ornithine transcarbamylase deficient patients, and 85% of the alleles seen in cystathionine beta-synthase (CBS)^3^ deficient patients [1]. Most disease causing missense mutations do not target key catalytic residues in enzymes but rather cause problems in protein folding resulting in non-functional proteins [2]. Treatments that could reverse these protein folding defects would be of great utility in the treatment of genetic disease.

Chemical chaperones are a diverse class of small molecules that can sometimes correct the protein folding defects inherent in mutant proteins. The best known example is the cystic fibrosis transmembrane conductance regulator (CFTR) ΔF508 mutation. This single amino acid deletion causes the CFTR protein to misfold and be degraded in the ER. Treatment of cells with glycerol has been shown to partially correct this defect [3]. Similar chaperone rescue has been observed for certain alleles in the aquaporin-2 and vasopressin-2 genes, mutations in which cause nephrogenic diabetes insipidus [4,5]. Although chemical chaperones have mostly been studied in the context of mutations that cause misfolding in the ER, there are reports involving *in vitro* enhancement of activity of mutant cytosolic enzymes [6,7].

CBS deficiency, or classic homocystinuria, is caused by mutations in the *CBS* gene and is the most common inherited disorder of sulfur metabolism [8]. Untreated CBS-deficient patients have extreme elevations in total plasma homocysteine (tHcy) and plasma methionine levels, associated with thrombosis, osteoporosis, mental retardation, psychiatric disorders, and dislocated lenses. To date, 140 different mutations have been described in CBS-deficient patients [9]. Over 85% of these mutations are missense mutations that would be predicted to encode full-length CBS protein. CBS is a 63 kDa protein and is normally functional as a homotetramer [10]. Studies in heterologous expression systems such as *Escherichia coli* and *Saccharomyces cerevisiae* indicate that the majority of missense mutant proteins are stable, but have greatly reduced specific activity [11,12]. The most common missense mutation, found in about one-quarter of all CBS patients, is the c.833T>C (I278T) allele [9]. Patients homozygous for this alteration tend to respond clinically to high dose pyridoxine, a precursor to the pyridoxal phosphate co-factor for the enzyme, but expression of this protein in mice, *E. coli*, or *S. cerevisiae* indicate that the alteration severely inhibits enzyme activity [13,14] possibly via misfolding [15].

Intriguingly, I278T and several other patient-derived missense mutations retain cryptic catalytic ability. Truncation or point mutation in the C-terminal regulatory domain is able to restore significant levels of enzyme activity to multiple different missense mutations located in the catalytic domain [16,17]. The ability to restore function by a *cis* acting second mutation suggested to us that it might be possible to restore function in *trans* via interaction of mutant CBS with a small molecule (i.e. drug). In order to pursue this possibility, we developed a simple yeast assay utilizing yeast expressing human I278T to screen for compounds that could rescue the growth defect. Surprisingly, we discovered in the process of library screening that the solvent compound dimethylsulfoxide (DMSO) by itself could rescue the I278T mutant. The work described here expands on this serendipitous discovery.

## Materials and methods

### Yeast strains and growth assays

Yeast strains and plasmids used in this study were created as previously described [18,19]. Synthetic complete media lacking cysteine (SC-cys) was made as described in [20] except that amino acids were added at 12× the stated level amount. SC + cys media were made by adding glutathione to the mixture at a final concentration of 30 μg/ml. All chemicals were purchased from Sigma–Aldrich (St. Louis, MO, USA).

**Fig. 1:**
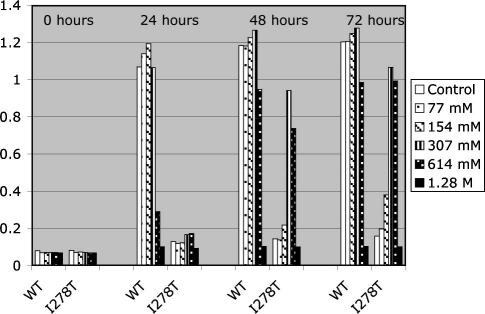
DMSO growth rescue of I278T expressing *S. cerevisiae*. Yeast strain WY35 (αleu2 ura3 ade2 trp1 cys4::LEU2) was transformed with an expression plasmid expressing either wild-type human CBS (pHCBS) or I278T CBS (pI278T). Yeast were diluted to an OD_600_ of 0.05 in SC-cys media with the indicated supplementation of DMSO in 384-well plates as described in ‘Materials and methods’. OD was measured at the indicated times using a plate reader. Data shown are the averages of three wells.

Yeast growth was monitored in two different assays. In the experiment shown in Fig. 1, yeast were grown in 384-well microtiter plates. Cells were diluted to a starting OD_600_ of approximately 0.03 and plated in wells containing 40 μl of media. Plates were incubated at 30 °C with no shaking and OD was measured using a Multiskan Ascent plate reader (Thermo Bioanalysis, Santa Fe, NM, USA). For all other experiments cells were grown in standard 15 ml borosilicate tubes containing 3 ml of media. In these experiments cells were grown at 30 °C with aeration. OD_600_ was determined using a Milton Roy Spectronic 601 spectrophotometer (Ivyland, PA, USA).

### Immunoblot and CBS enzyme analysis

Yeast extracts were prepared by mechanical lysis as previously described [16]. Protein concentration was determined by the Coomassie Blue protein assay reagent (Pierce) using BSA as a standard. For Western blot analysis under native conditions, extracts containing 20 μg of total protein were run on precast 4–15% gradient Criterion Tris–HCl gel (Bio-Rad) at 15 mA per gel and transferred to PVDF membrane as described [15]. Membranes were probed with a 1/5000 dilution of immunopurified rabbit anti-hCBS serum [11] followed by a 1/30,000 dilution of horseradish peroxidase-conjugated anti-rabbit secondary anti-body (Jackson ImmunoResearch Laboratories, West Grove, PA, USA). Signal was detected by chemiluminescence using SuperSignal West Pico Chemiluminescent Substrate (Amersham) and Chemigenius station (Syngene); signal was quantitated by GeneSnap software. For SDS-gels identical conditions were used with the exception of the presence of 3% sodium dodecyl sulfate (SDS) in the extraction buffer, 10 min heating at 100 °C prior loading and the presence of SDS in the electrophoresis running buffer.

Recombinant purified CBS and I278T CBS were prepared from *E. coli*-expressing GST-fusion proteins as previously described [21].

CBS enzyme activity was measured using a Biochrom 30 amino acid analyzer as previously described [22].

For the normalized activity shown in Fig. 4, we first determined the amount of CBS protein by densitometry using Alpha Ease™ image analysis software. We then normalized all enzyme activity to the wild-type enzyme by multiplying the enzyme activity by the amount of CBS in the wild-type untreated sample divided by the amount in each treated sample.

### In vitro transcription/translation

Wild-type and I278T human CBS cDNA were cloned into plasmid pTnT™ (Promega, www.promega.com) as previously described [21]. *In vitro* coupled transcription/translation was performed using the TnT™ Coupled Wheat Germ Extract Systems (Promega) according to manufacturer’s instructions.

## Results

### DMSO can rescue growth defect of I278T expressing S. cerevisiae

We first examined the ability of DMSO to allow growth of yeast expressing human I278T CBS (Fig. 1). Yeast expressing either wild-type human CBS (WT) or human I278T CBS (I278T) were grown in minimal media lacking cysteine and had DMSO added to the media at concentrations varying from 0 to 1.28 M. Yeast growth was measured by optical density (OD_600_). In the absence of DMSO we observed almost no growth of I278T, with the mean OD_600_ going from 0.08 to 0.15 after 72 h. In contrast, I278T grown in media containing either 307 mM or 614 mM DMSO showed significant growth. After 72 h I278T yeast grown in 307 mM DMSO had an OD_600_ of 1.07 compared to an OD_600_ of 1.25 for WT. Interestingly, there appeared to be a lag in the growth of I278T compared to WT. At 24 h there was only minimal stimulation of I278T growth, while WT had already reached saturation. We also found that high concentrations of DMSO (1.28 M) appeared to be toxic to both I278T and WT yeast. We failed to observe any rescue in CBS-null yeast cells (data not shown). These experiments show that DMSO can significantly rescue the cysteine auxotrophy of I278T expressing yeast and suggest that DMSO can somehow restore function to mutant human CBS.

### Chemical chaperone rescue of I278T and other missense mutations

**Fig. 2:**
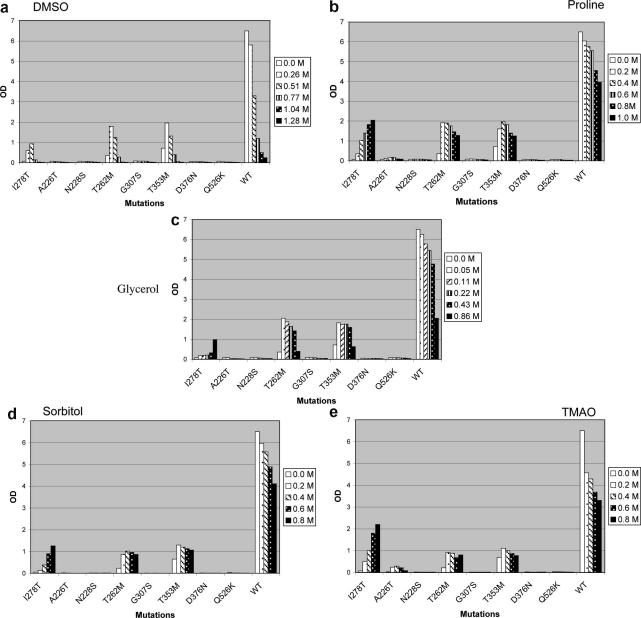
Chemical chaperone effect on growth of *S. cerevisiae* expressing mutant human CBS. Saturated cultures of yeast expressing the indicated mutant were diluted 1:1000 in SC-cys media with the indicated amount of chemical chaperone and grown in 15-ml tubes with aeration at 30 °C for 40 h. (a–e) Show the data for each of the five different chemical chaperones examined.

DMSO is a member of a class of low molecular weight compounds called chemical chaperones, osmolytes that can affect the process of protein folding [23]. We hypothesized that the rescue of I278T might occur by DMSO allowing the mutant I278T enzyme to fold into a more active conformation and thereby have sufficient enzyme activity to allow yeast to grow on cysteine-free media. If this was the case, other known osmolytes chemical chaperones might also rescue I278T expressing yeast. We examined growth rescue by four other known chemical chaperones; glycerol, proline, trimethylamine *N*-oxide (TMAO), and sorbitol. As shown in Fig. 2, all four of these compounds rescued growth of I278T significantly after 40 h, with the greatest amount of rescue occurring with TMAO and proline. Interestingly, all of the chaperones when given at high concentrations resulted in growth inhibition of yeast expressing wild-type CBS, indicating that chemical chaperones may adversely affect normal proteins.

We also determined if chemical chaperones could rescue other patient derived CBS missense mutations. In addition to I278T, we tested seven other missense mutations found in patients with CBS deficiency for growth with all five chemical chaperones. The mutations surveyed were A226T, N228S, T262M, G307S, T353M, D376N, and Q526K. All of these mutations with the exception of A226T are associated with pyridoxine non-responsive disease [19]. As shown in Fig. 2, the mutants fell into three classes. One class of mutations including N228S, G307S, D376N, and Q526K, were not rescuable by any of the chemical chaperones. A second class consisting of I278T, T262M, and T353M all showed significant rescue with all five chaperones tested. The third class contained a single member, A226T, and was characterized by exhibiting slight rescue with TMAO and proline, but not glycerol, DMSO, or sorbitol. From these studies we concluded that some, but not all, disease causing CBS alleles can have their residual enzyme activity enhanced by the addition of chemical chaperones. In addition, certain mutations can show chaperone specificity.

### Chaperone mixtures

**Fig. 3:**
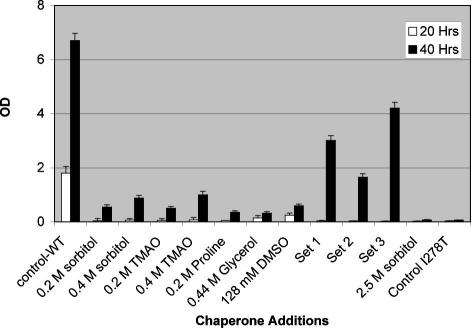
Comparison of the effectiveness of different chaperones and combinations of I278T expressing yeast. Yeast (WY35pI278T) were diluted and growth was assessed identically to Fig. 2. Chaperone Set 1 contains 0.2 M sorbitol, 0.2 M TMAO, 0.44 M glycerol, 0.128 M DMSO. Chaperone Set 2 contains 0.4 M sorbitol, 0.4 M TMAO, and 0.2 M proline. Chaperone Set 3 contains 0.4 M sorbitol, 0.44 M glycerol, and 0.128 M DMSO. The lane labeled Control-WT shows growth of WY35phCBS in SC-cys media. The lane labeled Control on the right is WY35pI278T in SC-cys media.

Since the chemical chaperones we used represented different categories of molecules (i.e. amino acids, sugars, oxides), we tried mixing different chaperones to determine if mixtures might enhance the rescuing effect. We examined growth rescue of the I278T mutant by three different chaperone mixtures. As shown in Fig. 3, all three mixtures rescued better than any of the individual chaperones alone when given at the same concentration. In the case of Mixtures 1 and 3 this increase was greater than the sum of the increases due to the individual chaperones. Mixture 3 produced the most growth rescue with the OD_600_ reaching 60% of the strain expressing wild-type human CBS. These findings show that combinations of different chaperones may be more effective than individual chaperones in promoting rescue of I278T CBS.

### Predicted change in solvent accessible surface area predicts chemical chaperone rescue

Chemical chaperones are osmolytes and are thought to affect protein folding by increasing water solvation of amino acids at the protein surface. Therefore, we examined how each of the eight mutations would be predicted to affect the solvent accessible surface area (ASA) of the CBS protein. To do this we compared the predicted solvent accessibility of wild type and mutant CBS proteins using NETASA, a neural network based prediction program that estimates the solvent accessibility of each amino acid based on the primary amino acid sequence [24]. From this analysis we obtained a window of 6–8 residues around each of the mutant residues that would be predicted to have a change in ASA. As shown in Table 1, five mutations would be predicted to cause an increase in mean ASA, while three changes would be predicted to cause a decrease in mean ASA. Interestingly, all three of the mutations causing a net decrease in mean ASA are rescuable by all four chemical chaperones tested, while the five mutations that result in increased ASA are not universally rescuable. These findings suggest that chemical chaperones may be most effective in restoring function to missense mutations that result in amino acids becoming more buried in the protein.

### Chemical chaperones cause enhancement of specific activity and tetramer formation of mutant CBS

**Fig. 4:**
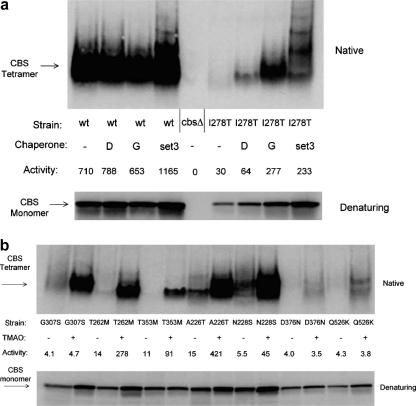
Chemical chaperones effect on wild-type and I278T CBS protein. (a) Yeast expressing either wild-type or I278T CBS were grown in SC + cys media with the indicated chemical chaperone overnight, and total lysates were examined for CBS by both native and denaturing PAGE followed by immunoblot as described in ‘Materials and methods’. Extracts were also examined for CBS activity as described in ‘Materials and methods’. D indicates 0.64 M DMSO. G is 0.44 M glycerol. Set 3 contains 0.4 M sorbitol, 0.44 M glycerol, and 128 mM DMSO. Activity is expressed in nmoles/mg total protein per hour. (b) Yeast expressing the indicated allele of CBS were grown in SC + cys media either in the presence (+) or in the absence of 1 M TMAO overnight. Total cell lysates were prepared and examined for CBS by both native and denaturing PAGE followed by immunoblot as described in ‘Materials and methods’. CBS enzyme activity was also determined for each lysate and results are expressed in nmoles/mg total protein per hour.

We next examined how chemical chaperones affected wild-type and mutant CBS protein expressed in *S. cerevisiae*. Yeast expressing either wild-type or I278T CBS were grown either in the absence or presence of chemical chaperones and then examined for CBS protein and activity. CBS protein was assessed both in denaturing gels to determine the total protein and in native gels to determine the amount of tetramer present. As shown in Fig. 4a, neither glycerol nor DMSO had a substantial effect on either wild-type CBS protein or activity, although Mixture 3 did cause a twofold increase in activity and the appearance of higher ordered multimers on native gels. In contrast, exposure of I278T expressing yeast to chemical chaperones resulted in a dramatic effect on CBS protein and activity. In the absence of chemical chaperones we found that cell extract from yeast expressing I278T had about 4% activity compared to yeast expressing WT CBS and almost no detectable tetramers. When chaperones were added to these cells we observed a large increase in both enzyme activity and the amount of tetramer. As expected, the most effective treatment was Mixture 3, resulting in an eightfold increase in CBS activity. Interestingly, this chaperone mixture promoted the formation of not just tetramers, but higher order multimers as well. From these results we conclude that yeast expressing I278T CBS in the presence of chemical chaperones had higher steady-state levels of CBS protein accompanied by an even larger increase in the amount of tetramer protein, resulting in increased CBS activity.

We also examined the effect of 1 M TMAO on CBS protein and activity produced from the seven additional mutants mentioned above. As shown in Fig. 4b, growth in TMAO resulted in increased tetramer content in all of the mutants. However, increased enzyme activity was only associated with four of the mutations, T262M, T353M, A226T, and N228S. From this result we conclude that chemical chaperones such as TMAO can promote proper protein folding and tetramer formation from a variety of different missense mutations. However, our data also show that although all of the missense mutations appear to affect tetramer formation, restoration of tetramers is not always sufficient to restore enzyme activity.

### Chaperone effect requires protein folding and is not a phenomena of S. cerevisiae

**Fig. 5:**
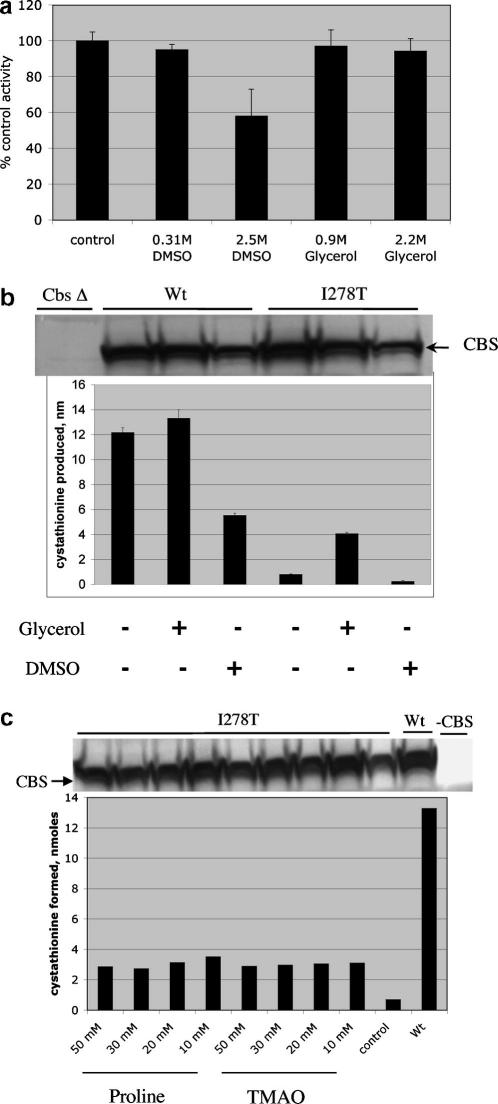
*In vitro* effects of chaperone addition. (a) DMSO and glycerol do not restore activity when added directly to purified recombinant I278T CBS. Recombinant I278T CBS was purified from *E. coli* as previously described [14]; 220 ng of purified enzyme was used in an *in vitro* reaction containing 10 mM serine, 10 mM l-homocysteine, and 100 mM AdoMet and the indicated amount of each chaperone. Results are expressed as a percentage of the control reaction (no chaperones). (b) Wild-type and I278T CBS were produced in an *in vitro* transcription/translation wheat germ extract either in the presence or absence of 0.9 M glycerol or 0.3 M DMSO, as described in ‘Materials and methods’. The presence of equal amounts of CBS protein produced was determined by western blot. Enzyme activity was measured in triplicate and error bars indicate standard deviation. Activity is expressed as amount of cystathionine produced in nanomoles. (c) Identical to (b) except that either proline or TMAO were added to the *in vitro* reactions.

We next determined if addition of chemical chaperones to purified I278T CBS could directly enhance enzyme activity. In these experiments we used recombinant purified I278T CBS from *E. coli* [21]. We failed to observe any stimulation of I278T enzyme activity with either the addition of glycerol or DMSO at two different concentrations (Fig. 5a). From these experiments we conclude that chemical chaperones directly added to folded I278T CBS did not increase enzyme activity.

The above result suggested that chaperone enhancement of enzyme activity of I278T requires that the chaperone must be present at the time the protein is initially folded. To test this idea we expressed wild-type and mutant CBS in an *in vitro* wheat germ transcription/translation system in which chemical chaperones were added. We found that addition of glycerol, proline, and TMAO all caused a four to eightfold increase in the specific activity of I278T CBS (Fig. 5b and c). We did not observe an increase when DMSO was added. In addition, we found that I278T purified and isolated from *E. coli* grown in 8% glycerol had a fourfold increase in specific activity compared to the enzyme isolated from cells grown in normal media (data not shown). From these experiments, we conclude that chemical chaperones can enhance I278T activity if they are present at the time of initial protein synthesis and production.

## Discussion

The vast majority of patients with clinical homocystinuria have missense mutations in the CBS gene. Here, we used a yeast functional assay to show that four of eight mutant CBS proteins found in homocystinuric patients could be partially rescued by the addition of chemical chaperones to the media. In the rescuable mutants, addition of chemical chaperone resulted in a significant increase in the specific activity of the produced mutant CBS enzyme. Importantly, chemical chaperones need to be present at the time the protein is produced and folded. We observe no stimulatory effect when the chaperones were added directly to already folded protein. This observation suggests that the chemical chaperones may promote the formation of properly formed early folding intermediates. There are reports that the chemical chaperones TMAO and sarcosine not only stabilize the native state but also early intermediates [25]. Such stabilization of the early intermediates would then lead to an acceleration in the folding rate.

Chemical chaperone rescue results in the stabilization of the CBS tetramer. All of the mutants examined in this study severely reduce the amount of stable tetramer. When chemical chaperones were added to the growth media significant increases in the amount of tetrameric CBS was observed. Surprisingly, most of the mutations tested did not have a large effect on the amount of total CBS protein present in the cells. This implies that the non-tetrameric form of CBS is stable. However, we failed to observe any monomeric or dimeric CBS in our native gels. Our inability to detect the non-tetrameric forms of the protein on native gels suggests that it may form some sort of unstable aggregate and therefore is part of the background “smear” that is present throughout each lane of the gel. It should also be pointed out that not all mutant proteins were rescued by growth in chaperones, despite the increased amount of tetramer present. G307S, D376N, and Q526K all had increased amounts of tetramers, but did not show increased activity. The most likely explanation for this is that these mutations have additional effects on the CBS protein. Examination of the crystal structure of truncated CBS protein (aa1–409, [26]) shows that the G307S mutation and the D376N mutation map very near the active site pocket and would likely disrupt access to the active site of the enzyme, whereas the suppressible mutations all map quite distant from the active site (data not shown).

In this study we used five different chemical chaperones, DMSO, glycerol, TMAO, proline, and sorbitol. These compounds are all osmolytes and therefore increase hydrostatic pressure. The current view about how osmolytes affect protein folding is that they force water molecules out of solution and “push” them to the protein surface. This increased hydration can then affect Gibbs free energy (G) of the protein-folding pathway such that the Δ*G* between the native and denatured state becomes larger thereby driving the equilibrium toward the native state [27]. We hypothesize that I278T and other mutant CBS enzymes can exist in one of two conformations, a properly folded active state (**A**) and an incorrectly folded inactive state (**I**). The **A** state would be characterized by the enzyme organizing in tetramer or higher order multimers, while in the **I** state the enzyme would be in some partially denatured and/or aggregated state. In aqueous conditions the **I** state is normally favored, but when chaperones are present the **A** state is now favored. Three mutants, I278T, T262M, and T353M responded well to all five of these agents. All three of these mutations would be predicted to make a region around the mutated amino acid more buried and less solvent accessible. Thus, it may be that the increased hydrostatic pressure caused by the osmolytes helps increase the solvation of these buried residues and that this causes the shift in equilibrium from the **I** to the **A** state.

Previously, we found that deletion or certain missense mutations in the C-terminal quarter of CBS could suppress some missense mutations located in the catalytic domain [16,17]. These findings are similar to other results that show that certain second site mutations can act as “global” suppressors of inactivating mutations. This was first observed by Shortle and Lin for staphylococcal nuclease, but has also been observed in several other enzymes including beta-lactamase, T4 lysozyme, and human p53 [28–31]. The current view of these findings is that the second site suppressors act by increasing protein stability and thus counteracting the effect of the original destabilizing mutation. In this study, we found a perfect correlation between mutations that were suppressible by chemical chaperones and mutations that were suppressible by truncation of the C-terminal regulatory domain. A hypothesis to explain this finding is that the C-terminal domain of CBS is a destabilizing influence and can promote misfolding of the catalytic domain when certain missense mutations are present. Absence of (or mutations in) the C-terminal domain reduce this destabilizing effect such that the catalytic domain can now fold properly. Similarly, chemical chaperones may increase the stability of the full length protein allowing proper folding and activity.

Our findings suggest novel clinical strategies to treat CBS deficiency and possibly other inborn errors of metabolism. In many inborn errors of metabolism the severity of the disease is related to the amount of residual enzyme activity present although this phenomenon is not universal. Raising the amount of residual activity even a small amount can have significant clinical benefits. It may be possible to increase this residual activity by ingestion of chemical chaperones. Glycerol, sorbitol and DMSO all have very high LD_50_ in rats ranging from 12.6 to 16 g/Kg body wt (http://physchem.ox.ac.uk/MSDS). In fact, osmolyte therapy using either glycerol or sorbitol is used routinely in humans. Medically, brain edema is commonly treated with IV solutions of glycerol or sorbitol at concentrations as high as 2 g/Kg body weight [32]. Oral glycerol at a dose of 1.2 g/Kg weight significantly improves endurance and is used by long distance athletes, such as marathoners and tri-athletes [33]. We observed stimulation of I278T expressing yeast with glycerol doses as low at 50 mM (0.5%) which would be equivalent to a 5 g/Kg solution. Thus, it may be possible to achieve glycerol concentrations *in vivo* sufficient to promote alternative folding of mutant CBS proteins and stimulate residual CBS activity. Future studies using CBS-deficient mouse models should be undertaken to test this hypothesis.

## Figures and Tables

**Table 1:** Predicted change in the accessible surface area (ASA) due to mutations

Mutation	Number of residues affected	Mean change in ASA (Å)
A226T	6	+7.1
N228S	8	+3.1
G307S	7	+5.9
Q526K	7	+7.5
D376N	7	+11.7
T353M	7	−6.8
T262M	7	−8.0
I278T	6	−4.4

## Footnotes

3 *Abbreviations used*: CBS, cystathionine beta-synthase; DMSO, dimethylsulfoxide; TMAO, trimethylamine-*N*-oxide; ASA, accessible surface area.
